# Association of Free Sugars Intake with Cardiometabolic Risk Factors among Japanese Adults: The 2016 National Health and Nutrition Survey, Japan

**DOI:** 10.3390/nu12123624

**Published:** 2020-11-25

**Authors:** Aya Fujiwara, Emiko Okada, Chika Okada, Mai Matsumoto, Hidemi Takimoto

**Affiliations:** 1 Department of Nutritional Epidemiology and Shokuiku, National Institute of Biomedical Innovation, Health and Nutrition, 1-23-1 Toyama, Shinjuku-ku, Tokyo 162-8636, Japan; fujiwaraay@nibiohn.go.jp (A.F.); okadae@nibiohn.go.jp (E.O.); chika-okada@umin.ac.jp (C.O.); m-matsumoto@nibiohn.go.jp (M.M.); 2 Department of Social and Preventive Epidemiology, Graduate School of Medicine, and School of Public Health, The University of Tokyo, 7-3-1 Hongo, Bunkyo-ku, Tokyo 113-0033, Japan

**Keywords:** free sugars, cardiometabolic risk factors, national survey, Japanese

## Abstract

The relationship between free sugars intake and cardiometabolic risk factors is unclear in Japanese adults. This cross-sectional study aimed to investigate this association using data from the 2016 National Health and Nutrition Survey, Japan. The percentage of energy intake from free sugars was estimated based on the 1-day weighed dietary record data of Japanese men (*n* = 4071) and women (*n* = 5794) aged ≥ 20 years. Associations between free sugars intake and cardiometabolic risk factors, including body mass index (BMI), waist circumference (WC), systolic and diastolic blood pressures, glycated haemoglobin (HbA1c) level and levels of serum total, low-density lipoprotein (LDL), and high-density lipoprotein (HDL) cholesterol, were investigated using linear regression and Dunnett’s test, with the lowest category of quartiles as a reference. After adjustment for potential confounding factors, free sugars intake was inversely associated with blood pressures (men only) and HDL-cholesterol level (both sexes) and positively associated with total-cholesterol level (women only) and LDL-cholesterol level (both sexes), whereas no association was observed for BMI, WC, and HbA1c level. This study identified both positive and inverse associations of free sugars intake with cardiometabolic risk factors in Japanese adults.

## 1. Introduction

Cardiovascular diseases are major contributors to the global burden of disease and the leading causes of death among non-communicable diseases, resulting in 17.6 million deaths and 300 million years of life lost in 2016 [1]. Due to this high disease burden, the identification of potentially modifiable factors influencing the pathogenesis of risk factors, such as diet, can help guide recommendations for the prevention of cardiovascular diseases.

As a potential dietary factor, free sugars intake may be related to adverse cardiometabolic outcomes [2,3,4]. This relationship could be mediated by weight gain via the contribution to energy intake, as shown in a previous meta-analysis [2]. Additionally, other pathways linking free sugars intake and increased cardiometabolic risk, independent of energy intake and weight gain, are likely, because free sugars intake has been positively associated with blood pressure and lipid profile independent of body weight in another meta-analysis [3]. Meanwhile, there is controversial result from another systematic review and meta-analysis [5]. Therefore, the effect of free sugars intake on cardiometabolic outcomes and further research is still required. Further, the majority of studies included in previous meta-analyses and systematic reviews conducted in Western countries have investigated the effects of free sugars from soft drinks [2,3,4]. One potential mechanism that may explain the effects of free sugars intake from soft drinks on cardiometabolic outcomes is its fructose content, as excess fructose intake can promote hepatic de novo lipogenesis to induce dyslipidaemia and insulin resistance and can increase blood pressure by the synthesis of uric acid [6]. One network meta-analysis indicated that fructose intake had a more adverse effect on low-density lipoprotein (LDL) cholesterol level, insulin resistance, and uric acid concentration compared to starch or glucose intake [7]. However, other systematic reviews and meta-analyses suggested that the adverse effect of fructose intake was rather mediated by excess energy intake than fructose per se [8,9]. Meanwhile, the association between fructose intake and blood pressure may be partly explained by increased salt intake rather than fructose intake, as the positive association between salt intake and total fluid consumption has been widely observed [10]. Additionally, more rapid absorption of free sugars from beverages than foods is more likely to lead to an acute increase in blood glucose and insulin levels. Thus, the consumption of large quantities of such beverages can promote insulin resistance [11] and inflammatory diseases [12]. Finally, as a widely consumed beverage, soft drinks (not free sugars per se) may act as a marker of an unhealthy lifestyle, including dietary habits [13,14], that can render individuals susceptible to adverse cardiometabolic outcomes.

Given the relatively lower intake of free sugars and soft drinks in the Japanese population than in Western populations [15], the association of free sugars intake and cardiometabolic risk factors would likely differ between Japan and Western countries. To our knowledge, a few studies have investigated an association between free sugars intake and cardiometabolic risk factors in Asian countries, including Japan [16,17]. A prospective study in Japanese adults has identified a positive association between free sugars intake and weight gain over a 10-year follow-up period in men; however, this association was not observed in women [16]. Meanwhile, an association between free sugars intake and other cardiometabolic risk factors, such as blood pressure, fasting blood glucose, and blood lipids, has been investigated in only adolescents in Japan and positive associations were observed for systolic blood pressure and fasting blood glucose [17]. However, such association has not been investigated in Japanese adults. Hence, this cross-sectional study aimed to investigate the relationship between free sugars intake and cardiometabolic risk factors among Japanese adults using 1-day dietary record data from the 2016 National Health and Nutrition Survey (NHNS), Japan.

## 2. Materials and Methods

### 2.1. Data Source and Analytic Sample

The NHNS is an annual nationwide nutrition survey. Local public health centres have conducted this survey since 1945 under the supervision of the Ministry of Health, Labour, and Welfare based on the Health Promotion Law in Japan. We performed this cross-sectional analysis based on data from the 2016 NHNS with permission from the Ministry of Health, Labour, and Welfare. We used data from the 2016 survey for the present analysis because of the significantly larger sample size than those in more recent surveys. Details of the survey have been previously described [18,19]. Briefly, using the 2010 national census, 475 census units were randomly sampled as survey areas; subsequently, 13 survey areas stricken by an earthquake or typhoon were excluded, and 462 areas were ultimately included in total. All non-institutionalised Japanese residents aged ≥ 1 year (as of 1 November 2016) in survey areas were eligible for the survey. Those excluded from the survey were: households wherein the heads were not Japanese, individuals without a self-selected diet, and individuals on a special diet (i.e., consuming full fluid diets or medicines only, mainly due to disease). The response rate of households was 44.4% (10,745 of 24,187 eligible households) for the final survey conducted between October and November 2016.

**Figure 1:**
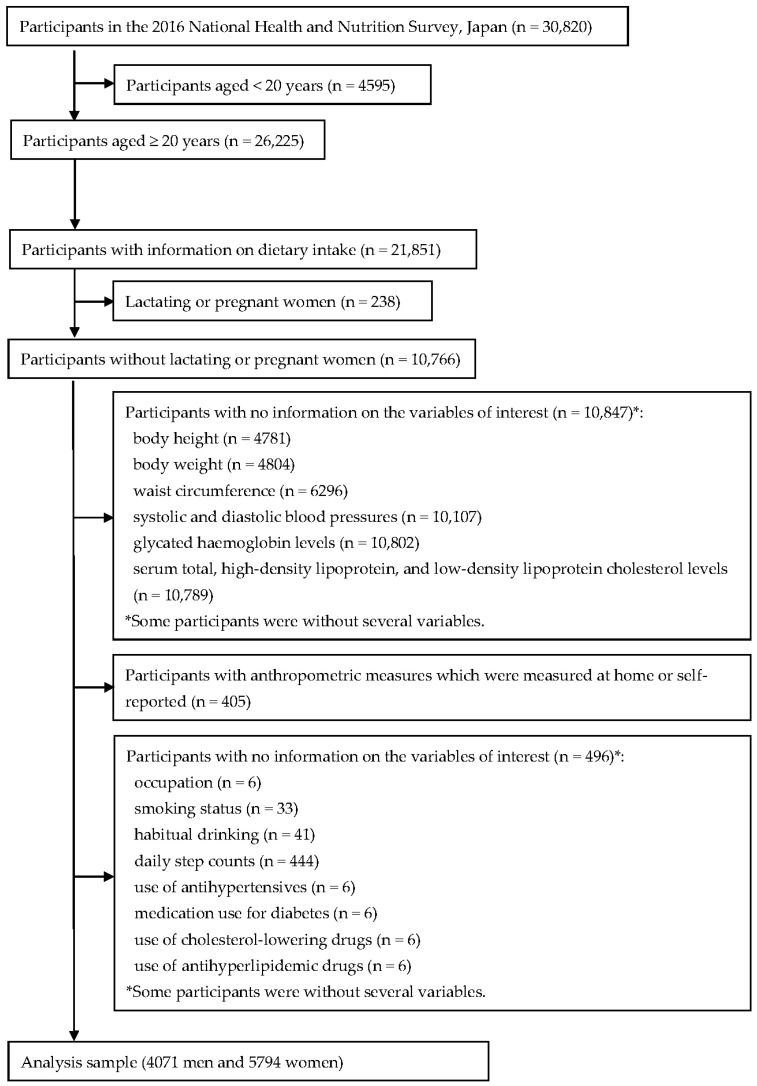
Flowchart of participants included in the present analysis.

Of the total participants in the 2016 NHNS (*n* = 30,820), 26,225 participants were aged ≥ 20 years (Figure 1). For the present analysis, we included all participants conducting the dietary survey (*n* = 21,851). Lactating or pregnant women were excluded (*n* = 238) because we considered that their dietary intake differed from that of non-lactating and non-pregnant women [20]. Participants without information on cardiometabolic risk factors, namely, body height, body weight, waist circumference (WC), systolic and diastolic blood pressures (SBP and DBP), glycated haemoglobin levels (HbA_1c_) and serum total, high-density lipoprotein (HDL), and LDL cholesterol levels were excluded (*n* = 10,847). Participants with anthropometric measures which were measured at home or self-reported were also excluded (*n* = 405). Additionally, we excluded participants with missing information on covariates, such as smoking status, habitual drinking, occupation, daily step counts, and the use of medications for hypertension, diabetes, and dyslipidaemia (*n* = 496, mainly due to daily step counts). The final sample included 9865 adults (4071 men and 5794 women).

This survey was conducted according to the guidelines dictated in the Declaration of Helsinki, and verbal informed consent was obtained from all individual participants. Under Article 33 of the Statistics Act, the Ministry of Health, Labour, and Welfare anonymised individual-level NHNS data and provided the first author with datasets for this study. The need for ethical review and institutional review board approval was waived for this analysis, according to the Ethical Guidelines of Epidemiological Research established by the Ministry of Education, Culture, Sports, Science, and Technology and the Ministry of Health, Labour, and Welfare [21], because only anonymised data were used.

### 2.2. Dietary Assessment

Dietary intake was assessed using 1-day weighed household dietary records, as described previously [18,19], and the analysis included data on all members of each household. Briefly, a diary for the dietary record was given to a primary meal preparer in the household (referred to as a record keeper) by a trained fieldworker (e.g., a registered dietitian), along with verbal and written instructions on the necessary procedure. All food and beverages (except for drinking water) consumed by household members on the survey day (except for Sundays, national holidays, and days with special events, such as wedding party or funeral) were recorded by the record keeper. For food items shared by household members from a single dish, approximate proportions of the food consumed by each member and the amounts of leftovers were recorded. When weighing was difficult (e.g., eating out), as much information as possible, such as estimated portion sizes and details of leftovers, was documented. Shortly after dietary recording (usually the next weekday), the diary was collected from the household by a trained fieldworker. If they found any missing or unclear information in the diary, they collected additional information from the record keeper. According to the NHNS study manual, the weights of foods and beverages consumed were estimated based on the portion sizes recorded using household measures, and food codes were assigned to all items (mainly based on the Standard Tables of Food Composition in Japan (STFCJ) [22]) by trained fieldworkers. Subsequently, the dietary records were reviewed by the trained fieldworkers at the local centre and the dietary intake data were inputted using software developed for the NHNS. Then, trained investigators merged the data to create an overall dietary dataset at the central office.

Intakes of foods, energy, and nutrients for participants were estimated from the household food consumption record, according to the STFCJ [23,24]. For shared dishes/foods, the approximate proportions consumed by each household member were determined, and food grouping was conducted based on the similarity in nutrient profiles and culinary usage. Free sugars were defined according to the World Health Organization (WHO) guideline [25] as sugars added by the manufacturer, cook, or consumer, and as the sugars naturally present in honey, syrups, and fruit juices and concentrates. Free sugars intake was estimated according to a comprehensive food composition database [15] for common Japanese food items included in the STFCJ [23,24], which was recently developed using a published step-wise method [26,27].

The ability of the household dietary record for estimating dietary intake at the individual-level has been previously validated in the Japanese population [28]. Briefly, the dietary intake of young women (aged about 20 years) estimated based on a 1-day household dietary record conducted by the women’s mothers (mean age, 49 years) was compared with those conducted by the young women themselves (*n* = 32). The mean differences between the intakes of energy, protein, fat, and carbohydrate estimated by the two methods were 6.2%, 5.7%, 6.7%, and 6.3%, respectively. The Pearson correlation coefficients for energy and these nutrients were 0.90, 0.89, 0.91, and 0.90, respectively [28].

### 2.3. Assessment of Cardiometabolic Risk Factors and Covariates

For most participants (>95%), trained fieldworkers (e.g., public health nurses or medical doctors) conducted anthropometric measurements in standardised procedures. Body height and weight of the participant were measured to the nearest 0.1 cm and 0.1 kg respectively, in light clothes and barefoot. WC was measured to the nearest 0.5 cm at the level of the umbilicus and the end of normal respiration, with the participant standing erect with the arms by the sides and the feet together. Otherwise, other household members took these measurements at home or the values were self-reported. BMI was calculated as dividing weight (kg) by square of height (m^2^). Trained fieldworkers measured SBP and DBP on the right arm by a standard mercury sphygmomanometer after the participant had seated and rested for ≥5 min. A second measurement was performed 1–2 min after the first measurement and the mean of the two measurements was used for the present analysis. HbA_1c_ and serum total, LDL-, and HDL-cholesterol concentrations were measured using non-fasting blood samples at a commercial laboratory [18,19,29].

Information on sex, age, smoking status, habitual drinking, occupation, and medication use for hypertension, diabetes, and dyslipidaemia (cholesterol-lowering and antihyperlipidemic drugs) was collected by a self-administered questionnaire. Daily step counts were measured by participants using a pedometer on any one day other than Sundays and national holidays.

### 2.4. Statistical Analysis

Statistical analyses were separately performed by sex using SAS statistical software, version 9.4 (SAS Institute Inc., Cary, NC, USA). All reported *p*-values were two-tailed, and *p* < 0.05 was considered statistically significant. Descriptive data are presented as means and standard deviations (SD) for continuous variables and as percentages of participants for categorical variables. To minimise the influence of dietary misreporting [30] and account for differences in dietary intake due to varying body sizes and energy requirements [31], free sugars intake and the diet quality score were energy-adjusted using the density method. In addition to total energy-adjusted intake (i.e., % of energy intake, %E), the mean contribution of each food group (%) to free sugars intake was calculated. Based on the distribution of free sugars intake, participants were divided into quartiles. The difference in basic characteristics according to the free sugars intake category was examined using a linear trend test (for continuous variables) or a Mantel-Haenszel chi-square test (for categorical variables). The adjusted mean intakes and standard errors (SE) for cardiometabolic risk factors were estimated for each category of free sugars intake using general linear models. The differences among free sugars intake categories were examined with the Dunnett’s test using the lowest category as a reference with adjustment for potential confounding factors, including age (20–29, 30–39, 40–49, 50–59, 60–69, 70–79, or ≥80 years), smoking status (never, past, or current), habitual drinking (yes or no), occupation (professional/manager, sales/service/clerical, security/transportation/labour, or not in paid employment), daily step counts (in tertiles), and intakes of energy (kcal, continuous), fat (%E, continuous), and dietary fibre (g/1000 kcal, continuous). Except for BMI and WC, further adjustment was conducted for medication use (no or yes: antihypertensives for blood pressure, antidiabetic drugs for HbA_1c_ level, and cholesterol-lowering and antihyperlipidemic drugs for blood cholesterol level) and BMI (kg/m^2^, continuous). Linear regression was performed using the median value of each category of free sugars intake as a continuous variable, with adjustment for the same variables used in the Dunnett’s test.

### 2.5. Sensitivity Analysis

Sensitivity analysis was performed, excluding the participants with misreported energy intakes. Previous studies have reported that selective underreporting of the intakes of confectioneries and soft drinks was common among participants with underreporting of energy intake [32,33]. Furthermore, participants in the lowest free sugars intake were likely to underreport energy intake [27,34,35]. In this study, misreporting of energy intake was defined as the ratio of reported energy intake to the basal metabolic rate (BMR) (i.e., the Goldberg cut-off [36]), as described previously [37,38]. Briefly, BMR was estimated using the sex- and age-specific equations developed for the Japanese population based on age, height, and body weight [39,40]. Participants with an energy intake to BMR ratio of 0.87–2.75 were defined as plausible reporters for energy intake by the Goldberg cut-off for 1-day dietary record data and the physical activity level for a sedentary lifestyle (i.e., 1.55, because an objective measurement of physical activity was not conducted in this survey) [30].

## 3. Results

### 3.1. Free Sugars Intake and Food Sources

The mean energy-adjusted intakes (SD) of free sugars intake were 4.8 (3.9) %E in men and 5.8 (4.2) %E in women (Table 1). Major contributors of free sugars intake (≥10%) were sugars and jams (30.1% in men and 29.8% in women), seasonings (24.4% and 21.3%), confectioneries (17.0% and 23.0%), and soft drinks (11.3% and 9.4%).

### 3.2. Characteristics of the Participants

The mean (SD) age of participants was 60.7 (16.1) years in men and 59.9 (15.8) years in women. The basic characteristics of participants according to free sugars intake are shown in Table 2 (for men) and Table 3 (for women). Participants having higher free sugars intake were more likely to be older, not in paid employment, and non-drinkers, use cholesterol-lowering drugs, and have a higher energy intake. Additionally, men with higher free sugars intake were more likely to have never smoked, whereas women with higher free sugars intake were less likely to use medication for diabetes.

### 3.3. Association Between Free Sugars Intake and Cardiometabolic Risk Factors

Table 4 shows the association between free sugars intake and cardiometabolic risk factors. After adjustment for potential confounding factors, including energy intake and BMI, free sugars intake was inversely associated with SBP, DBP, and HDL-cholesterol levels in men (all *p* for trend ≤ 0.04), with significantly lower mean SBP and HDL-cholesterol levels in the highest quartile (*p* = 0.008 and *p* < 0.0001, respectively) and the second-highest quartile (*p* = 0.007 for HDL-cholesterol level only) than in the lowest quartile. Further, free sugars intake was positively associated with LDL-cholesterol level in men (*p* for trend = 0.0003), with significantly higher mean LDL-cholesterol level in the highest and the second-highest quartiles than in the first quartile (*p* = 0.0009 and *p* = 0.008, respectively). In women, the inverse association of free sugars intake with SBP and DBP was not observed. Meanwhile, free sugars intake was also inversely associated with HDL-cholesterol level (*p* for trend = 0.02) in women, with significantly lower mean HDL-cholesterol level (*p* = 0.02), and positively associated with total- and LDL-cholesterol levels (*p* for trend = 0.04 and *p* for trend = 0.002, respectively), with significantly higher mean LDL-cholesterol level in the highest quartile than in the lowest quartile (*p* = 0.005). For BMI, WC, and HbA1c level, no association was observed in both sexes. The analysis without adjustment for BMI (except for BMI and WC) and energy intake did not substantially change the results (data not shown).

### 3.4. Sensitivity Analysis

In total, 158 (3.9%) men and 324 (5.6%) women were excluded from the sensitivity analysis due to misreported information. However, the results after participant exclusion were not substantially different from those of the primary analysis (data not shown).

## 4. Discussion

To our knowledge, this is the first study to investigate the association between free sugars intake and cardiometabolic risk factors in a nationally representative sample of a non-Western population. The present results identified that higher free sugars intake is inversely associated with blood pressure in men and adversely associated with blood cholesterol levels in both sexes, independent of energy intake and BMI. Meanwhile, no association was observed between free sugars intake and obesity measures and HbA_1c_ level.

Free sugars intake was not significantly associated with BMI and WC in this cross-sectional study, even without adjustment for energy intake. A previous meta-analysis of randomised control trials and prospective cohort studies has reported that free sugars intake was positively associated with bodyweight via an increase in energy intake [2]. Meanwhile, a previous cross-sectional study in Australian adults did not identify a significant association between free sugars intake and BMI and WC without adjustment for energy intake [41]. Although there were differences in study populations and confounding factors considered between this study and studies included in the meta-analysis, the relatively low mean free sugars intake (<6%E) and a cross-sectional design in the present population might not be sufficient to identify the association with BMI (and thus WC). A previous cohort study in Japanese adults with relatively lower mean free sugars intake (29.4 g/day in men and 28.6 g/day in women) has shown a positive association of free sugars intake with weight gain during a 10-year follow-up period in men, unlike women, even after adjustment for energy intake [16]. The authors mentioned the differences between men and women, in the change in free sugars intake and other factors influencing weight gain during the follow-up period. However, the significant association after adjustment for energy intake suggested that it may not be due to an increase in energy intake from free sugars. Therefore, in terms of energy-providing nutrients, free sugars might not substantially contribute to obesity measures in Japanese adults who have relatively low intake.

On the contrary, free sugars intake showed an inverse association with blood pressure in men. A similar association has equally been identified in the aforementioned Australian study, although adjustment for energy intake and BMI was not conducted [41]. In contrast, another meta-analysis has reported a positive association between free sugars intake and blood pressure, independent of energy intake and BMI [3]. The majority of studies included in the meta-analysis have investigated the effects of free sugars from soft drinks [3], and previous findings suggested that liquid sugars influence blood pressure [42,43]. Therefore, the disparity in the results is probably because of lower fructose intake [6] in this study due to its low soft drink contribution to free sugars intake (11.3% in men and 9.4% in women) than those of Western countries (10.2–31.8% (as added sugars) in Australia [44] and 15% in the UK [45]). In contrast, the association of free sugars intake with blood pressure was not significant in women. Although women had higher mean intakes of free sugars intake than men, the minimal difference (i.e., 1.0%E) was likely insufficient to produce the difference in the association. Moreover, because the contribution of soft drinks to free sugars intake was somewhat higher in men than women (i.e., 2.0%), free sugars intake from soft drinks could be similar in both sexes. Therefore, this discrepancy might be due to hormonal and biological differences as well as different levels of dietary misreporting (not free sugars intake per se) between sexes.

For HbA_1c_ level, no significant association was observed in this study, contrary to previous findings on blood glucose and insulin levels from studies investigating the influence of liquid sugars [4,46]. However, a previous cross-sectional study in British adults has reported positive associations of free sugars intake with fasting insulin level and homeostasis model assessment of insulin resistance and no association with blood glucose and HbA_1c_ [42]. Besides the low mean intake and the sources of sugars, differences in measures for cardiometabolic factors could influence the discrepancy in the results.

Meanwhile, higher free sugars intake was similarly associated with adverse profiles of blood cholesterol (i.e., lower HDL-cholesterol level and higher total- and LDL-cholesterol levels), in line with the findings of a previous meta-analysis reporting the adverse association between free sugars intake and blood lipids [3]. Because the mean intake in this study was considered low, this association was unlikely due to fructose intake [6,7]. However, intakes of total sugars from both beverages and foods, along with free sugars, were inversely associated with HDL-cholesterol levels in the previous British cross-sectional study [42]. Therefore, the relationship between free sugars intake and blood cholesterol levels might not be influenced by differences in study design and the type of sugars. Only further analysis investigating the influence of each sugar type (e.g., fructose) can elucidate the differing relationships between the intakes of free sugars and cardiometabolic risk factors in different populations.

Several limitations of this study should be mentioned. First, due to the cross-sectional design, we could not assess causality owing to the uncertain temporality of the association. A prospective study would provide a better understanding of the effect of free sugars intake on cardiometabolic risk factors. Second, despite the fact that the NHNS aimed to include a nationally representative sample of the non-institutionalised population of Japan, only 44.4% of the sampled households took part in the survey. In addition, no information on the characteristics of households that refused to participate was available, and the exact response rate at the individual-level was not determined [18]. Furthermore, the participants included in the present analysis (*n* = 10,248) had somewhat different characteristics from those excluded from the analysis because of missing information (*n* = 11,365). Those excluded were more likely to be male, young, in paid employment, current smokers, and have a lower BMI and energy intake (*p* ≤ 0.006) than the study participants. Therefore, a degree of selection bias cannot be ruled out.

Third, since free-living individuals have the day-to-day variation in their dietary intake, the 1-day weighed household dietary records are unlikely to represent the habitual dietary intake of individual participants. Although dietary assessment was not conducted on Sundays or national holidays, there was no information for the day selected for recording [18]. Furthermore, dietary intake possibly differs among seasons because the surveys were carried during a specific time window [47,48]. These limitations tend to result in bias among the assessment of average dietary intake and may affect the accuracy of the present finding. The use of multiple-day dietary data covering all seasons and days of the week or a validated food frequency questionnaire would be ideal solutions to resolve these issues in this study. Unfortunately, no nationwide representative dietary survey was conducted based on multiple-day dietary assessment methods. The feasibility of these solutions should be examined for the NHNS in the future.

Fourth, misreporting of dietary intake is commonly available in dietary surveys and may hinder the accuracy of the relationship between dietary intake and the variables of interest. For example, participants with the lowest free sugars intake tend to underreport energy intake [27,34,35], whereas participants underreporting energy intake were more likely to underreport confectionery and soft drink consumption [32,33]. However, the results of the sensitivity analysis did not substantially differ from those of the principal analysis in the present analysis (data not shown). Therefore, the influence of misreporting on the present findings was not significant and would not change the conclusion on the association between free sugars intake and nutrient intake. Additionally, the previous study has examined the utility of the household dietary record for estimating the dietary intake of individual participants only in young women who were not the participants in the NHNS [28]. Therefore, the utility in the participants in the NHNS, especially older women or men, remains unclear. Finally, although we performed adjustment for a variety of potential confounding variables, including dietary intake, energy intake, and BMI, residual confounding may remain.

## 5. Conclusions

This study identified the inverse associations of free sugars intake with blood pressure and the adverse association with blood cholesterol levels in Japanese adults, independent of energy intake and BMI. The present findings provide information about the consumption of free sugars that help in developing public health policies, such as the development of the Dietary Reference Intake for Japanese [49]. Despite its inverse association with blood pressure and no association with obesity measures and blood glucose level, excessive free sugars intake should not be recommended because it may influence weight status by contributing to energy intake, similarly to other energy-providing nutrients, and may induce dental caries. Further surveys investigating the influence of each sugar, such as fructose, in prospective design can help to clarify the mechanisms underlying the difference in associations between free sugars intake and cardiometabolic risk factors among the previous and the present study populations.

## Figures and Tables

**Table 1:** Contribution (%) of each food group to free sugars intake in Japanese men (*n* = 4071) and women (*n* = 5794): the 2016 National Health and Nutrition Survey, Japan.

	Men		Women	
	Mean	SD	Mean	SD
Intake (%E)	4.8	3.9	5.8	4.2
Contribution (%) ^1,2^				
Bread	0.2	2.3	0.3	2.5
Noodles	0.6	4.5	0.3	2.6
Other grain products	0.4	3.1	0.7	4.5
Potatoes	0.0	0.1	0.0	0.4
Sugars and jams	30.1	27.4	29.8	26.6
Pulses and nuts	1.2	6.7	1.6	7.3
Vegetables ^3^	2.2	7.0	1.6	5.9
Fruits	0.3	2.8	0.3	2.6
Fish and shellfish	5.1	11.5	4.2	10.1
Meats	2.0	6.7	1.4	5.1
Eggs	0.1	0.7	0.1	0.9
Dairy products	2.8	9.7	4.1	11.7
Fat and oil	0.4	2.9	0.3	2.8
Confectioneries	17.0	25.9	23.0	28.0
Fruit and vegetable juices	1.0	7.4	1.0	7.1
Alcoholic beverages	0.8	5.9	0.5	4.7
Soft drinks ^4^	11.3	23.4	9.4	20.5
Seasonings ^5^	24.4	24.8	21.3	22.1

SD, standard deviation; %E, percent of energy. ^1^ Estimated based on the intake of consumers: 4066 men and 5786 women. ^2^ Rice and grains and unsweetened tea and coffee were excluded due to the no contribution to free sugars intake. ^3^ Including mushrooms and seaweeds. ^4^ Including soda, sports drinks, fruit drinks, milk beverages, pre-sweetened coffee, and ama-zake (Japanese traditional sweet beverages made from rice koji). ^5^ Including mirin (Japanese traditional sweet seasoning made from rice, rice koji, and distilled alcohol), non-oil dressing, and other sauces.

**Table 2:** Basic characteristics of Japanese men (*n* = 4701) according to free sugars intake: the 2016 National Health and Nutrition Survey, Japan.

	Quartile 1 (*n* = 1017)		Quartile 2 (*n* = 1018)		Quartile 3 (*n* = 1018)		Quartile 4 (*n* = 1018)		*p* ^1^
Free sugars intake (%E), mean ± SD	1.2	0.5	289	0.5	5.0	0.8	10.0	3.9	<0.0001
Age (years), mean ± SD	58.9	15.6	61.4	15.5	62.0	15.9	60.4	17.0	0.16
Age category (years), n (%)									
20–29	36	(3.5)	35	(3.4)	38	(3.7)	68	(6.7)	0.02
30–39	96	(9.4)	88	(8.6)	89	(8.7)	87	(8.6)	
40–49	178	(17.5)	117	(11.5)	111	(10.9)	115	(11.3)	
50–59	150	(14.7)	129	(12.7)	129	(12.7)	118	(11.6)	
60–69	284	(27.9)	312	(30.6)	288	(28.3)	277	(27.2)	
70–79	200	(19.7)	239	(23.5)	257	(25.2)	251	(24.7)	
≥80	73	(7.2)	98	(9.6)	106	(10.4)	102	(10.0)	
Occupation, n (%)									
Professional/manager	219	(21.5)	199	(19.5	191	(18.8)	177	(17.4)	0.003
Sales/service/clerical	172	(16.9)	170	(16.7)	155	(15.2)	175	(17.2)	
Security/transportation/labour	296	(29.1)	284	(27.9	297	(29.2)	274	(26.9)	
Not in paid employment	330	(32.4)	365	(35.9)	375	(36.8)	392	(38.5)	
Smoking status, n (%)									
Never	542	(53.3)	577	(56.0)	589	(57.9)	596	(58.5)	0.02
Past	180	(17.7)	202	(19.8)	200	(19.6)	159	(15.6)	
Current	295	(29.0)	239	(23.5)	229	(22.5)	263	(25.8)	
Habitual drinking, n (%) ^2^									
Yes	722	(71.0)	707	(69.4)	669	(65.7)	561	(55.1)	<0.0001
Daily step counts, n (%) ^3^									
Low	347	(34.1)	338	(33.2)	325	(31.9)	347	(34.1)	0.35
Middle	353	(34.7)	336	(33.0)	344	(33.8)	324	(31.8)	
High	317	(31.2)	344	(33.8)	349	(34.3)	347	(34.1)	
Use of medication, n (%)									
Antihypertensives	312	(30.7)	352	(34.6)	346	(34.0)	323	(31.7)	0.70
For diabetes	97	(9.5)	125	(12.3)	94	(9.2)	89	(8.7)	0.20
Cholesterol-lowering drugs	104	(10.2)	122	(12.0)	152	(14.9)	140	(13.8)	0.002
Antihyperlipidemic drugs	54	(5.3)	63	(6.2)	70	(6.9)	49	(4.8)	0.81
Energy intake (kcal), mean ± SD	2110	574	2189	564	2265	531	2247	571	<0.0001
Fat intake (%E), mean ± SD	26.2	7.8	26.5	7.9	25.7	6.9	25.1	7.4	<0.0001
Dietary fibre intake (g/1000 kcal), mean ± SD	6.8	2.7	7.3	2.8	7.6	2.8	7.4	2.9	<0.0001

SD, standard deviation; %E, percent of energy. ^1^ For continuous variables, a linear regression was used with the median value of each category of free sugars intake (1.2, 2.8, 5.0, and 8.9%E) as a continuous variable; for categorical variables, Mantel-Haenszel chi-square test was used. ^2^ Participants with drinking habit (yes) were defined as those who responded yes to drinking alcohol every day to 1–3 time(s)/week, and participants without habitual drinking (no) were defined as those who responded rarely, quit, or not drinking alcohol. ^3^ Classified into tertiles: ≤4435 steps for low, 4439–7689 steps for middle, and ≥7691 steps for high.

**Table 3:** Basic characteristics of Japanese women (*n* = 5794) according to free sugars intake: the 2016 National Health and Nutrition Survey, Japan.

	Quartile 1 (*n* = 1448)		Quartile 2 (*n* = 1449)		Quartile 3 (*n* = 1449)		Quartile 4 (*n* = 1448)		*p* ^1^
Free sugars intake (%E), mean ± SD	1.7	0.7	3.9	0.6	6.2	0.8	11.3	4.4	<0.0001
Age (years), mean ± SD	59.2	15.6	59.6	15.6	60.5	15.5	60.3	16.3	0.03
Age category (years), n (%)									
20–29	48	(3.3)	50	(3.5)	48	(3.3)	55	(3.8)	0.02
30–39	137	(9.5)	140	(9.7)	130	(9.0)	144	(9.9)	
40–49	239	(16.5)	208	(14.4)	189	(13.0)	193	(13.3)	
50–59	228	(15.7)	237	(16.4)	235	(16.2)	197	(13.6)	
60–69	400	(27.6)	385	(26.6)	400	(27.6)	398	(27.5)	
70–79	272	(18.8)	318	(21.9)	308	(21.3)	303	(20.9)	
≥80	124	(8.6)	111	(7.7)	139	(9.6)	158	(10.9)	
Occupation, n (%)									
Professional/manager	170	(11.7)	188	(13.0)	155	(10.7)	155	(10.7)	0.04
Sales/service/clerical	401	(27.7)	403	(27.8)	376	(25.9)	387	(26.7)	
Security/transportation/labour	146	(10.1)	129	(8.9)	128	(8.8)	139	(9.6)	
Not in paid employment	731	(50.5)	729	(50.3)	790	(54.5)	767	(53.0)	
Smoking status, n (%)									
Never	1305	(90.1)	1317	(90.9)	1332	(91.9)	1313	(90.7)	0.37
Past	41	(2.8)	60	(4.1)	40	(2.8)	47	(3.2)	
Current	102	(7.0)	72	(5.0)	77	(5.3)	88	(6.1)	
Habitual drinking, n (%) ^2^									
Yes	478	(33.0)	461	(31.8)	440	(30.4)	432	(29.8)	0.04
Daily step counts, n (%) ^3^									
Low	489	(33.8)	461	(31.8)	459	(31.7)	523	(36.1)	0.13
Middle	463	(32.0)	491	(33.9)	519	(35.8)	458	(31.6)	
High	496	(34.3)	497	(34.3)	471	(32.5)	467	(32.3)	
Use of medication, n (%)									
Antihypertensives	375	(25.9)	380	(26.2)	404	(27.9)	384	(26.5)	0.50
For diabetes	97	(6.7)	76	(5.2)	58	(4.0)	59	(4.1)	0.0004
Cholesterol-lowering drugs	232	(16.0)	256	(17.7)	289	(19.9)	268	(18.5)	0.03
Antihyperlipidemic drugs	52	(3.6)	59	(4.1)	49	(3.4)	66	(4.6)	0.33
Energy intake (kcal), mean ± SD	1675	442	1789	432	1801	432	1829	482	<0.0001
Fat intake (%E), mean ± SD	28.5	8.1	28.6	7.1	27.9	7.2	26.4	7.4	<0.0001
Dietary fibre intake (g/1000 kcal), mean ± SD	8.6	3.2	8.9	3.1	8.9	3.3	8.5	3.0	0.19

SD, standard deviation; %E, percent of energy. ^1^ For continuous variables, a linear regression was used with the median value of each category of free sugars intake (1.8, 3.9, 6.1, and 10.0%E) as a continuous variable; for categorical variables, Mantel-Haenszel chi-square test was used. ^2^ Participants with drinking habit (yes) were defined as those who responded yes to drinking alcohol every day to 1–3 time(s)/week, and participants without habitual drinking (no) were defined as those who responded rarely, quit, or not drinking alcohol. ^3^ Classified into tertiles: ≤4123 steps for low, 4128–6997 steps for middle, and ≥6998 steps for high.

**Table 4:** Cardiometabolic risk factors of Japanese men (*n* = 4071) and women (*n* = 5794) according to free sugars intake: the 2016 National Health and Nutrition Survey, Japan.

	Men									Women								
	Quartile 1 (*n* = 1017)		Quartile 2 ^1^ (*n* = 1018)		Quartile 3 ^1^ (*n* = 1018)		Quartile 4 ^1^ (*n* = 1018)		*p* ^2^	Quartile 1 (*n* = 1448)		Quartile 2 ^1^ (*n* = 1449)		Quartile 3 ^1^ (*n* = 1449)		Quartile 4 ^1^ (*n* = 1448)		*p* ^2^
BMI (kg/m^2^)																		
Crude	24.1	0.1	24.0	0.1	23.7	0.1	24.0	0.1	0.50	22.6	0.1	22.6	0.1	22.7	0.1	22.6	0.1	0.80
Adjusted ^3^	24.0	0.1	24.0	0.1	23.7	0.1	24.0	0.1	0.98	22.6	0.1	22.7	0.1	22.7	0.1	22.5	0.1	0.42
WC (cm)																		
Crude	87.0	0.3	86.9	0.3	86.2	0.3	87.0	0.3	0.82	81.6	0.3	82.1	0.3	82.4	0.3	82.2	0.3	0.17
Adjusted ^3^	87.0	0.3	86.8	0.3	86.2	0.3	87.2	0.3	0.65	81.8	0.3	82.2	0.3	82.3	0.3	82.0	0.3	0.67
SBP (mmHg)																		
Crude	135	1	135	1	135	1	132	1 *	0.0005	129	0	128	0	128	0	128	0	0.98
Adjusted ^4^	135	0	134	0	135	0	133	0 *	0.0003	129	0	128	0	128	0	128	0	0.16
DBP (mmHg)																		
Crude	82	0	82	0	81	0 *	80	0 *	<0.0001	77	0	77	0	77	0	77	0	0.89
Adjusted ^4^	82	0	81	0	81	0	81	0	0.04	77	0	77	0	77	0	77	0	0.89
HbA_1c_ (%)																		
Crude	5.7	0.0	5.8	0.0 *	5.8	0.0	5.8	0.0	0.58	5.7	0.0	5.7	0.0	5.7	0.0	5.7	0.0	0.10
Adjusted ^5^	5.8	0.0	5.8	0.0	5.8	0.0	5.8	0.0	0.78	5.7	0.0	5.7	0.0	5.7	0.0	5.7	0.0	0.40
TC (mg/dl)																		
Crude	197	1	197	1	197	1	195	1	0.14	207	1	208	1	208	1	208	1	0.52
Adjusted ^6^	196	1	197	1	197	1	197	1	0.43	207	1	207	1	208	1	209	1	0.04
HDL-C (mg/dl)																		
Crude	58	0	57	0	57	0	54	0 *	<0.0001	68	0	67	0	66	0	66	0 *	0.002
Adjusted ^6^	58	0	57	0	56	0*	55	0 *	<0.0001	68	0	66	0	66	0	66	0 *	0.02
LDL-C (mg/dl)																		
Crude	115	1	115	1	116	1	117	1	0.04	119	1	120	1	121	1	121	1	0.15
Adjusted ^6^	113	1	115	1	117	1 *	118	1 *	0.0003	119	1	120	1	121	1	122	1 *	0.002

All values are adjusted means and standard errors unless otherwise indicated. BMI, body mass index; WC, waist circumference; SBP; systolic blood pressure; DBP, diastolic blood pressure; HbA1c, glycated haemoglobin; TC, total cholesterol; HDL-C, HDL-cholesterol; LDL-C, LDL-cholesterol; %E, percent of energy. ^1^ Dunnett’s test was conducted using the lowest category as a reference: * *p* < 0.05. ^2^ A linear regression was conducted with the median value of each category of free sugars intake (1.2, 2.8, 5.0, and 8.9%E for men and 1.8, 3.9, 6.1, and 10.0%E for women). ^3^ Adjustment was made for age category (20–29, 30–39, 40–49, 50–59, 60–69, 70–79, ≥80), smoking status (never, past, or current), habitual drinking (yes or no), occupation (professional/manager, sales/service/clerical, security/transportation/labour, or not in paid employment), daily step counts (low, middle, high), and intakes of energy (kcal, continuous), fat (%E, continuous), and dietary fibre (g/1000 kcal, continuous). ^4^ Additionally to variables adjusted in the analysis of BMI and WC, adjustment was made for BMI (kg/m^2^, continuous) and antihypertensives use (yes or no). ^5^ Additionally to variables adjusted in the analysis of BMI and WC, adjustment was made for BMI (kg/m^2^, continuous) and medication use for diabetes (yes or no). ^6^ Additionally to variables adjusted in the analysis of BMI and WC, adjustment was made for BMI (kg/m^2^, continuous) and use of cholesterol-lowering and antihyperlipidemic drugs (yes or no).
